# Therapeutic implications of cancer-associated fibroblast heterogeneity: insights from single-cell and multi-omics analysis

**DOI:** 10.3389/fimmu.2025.1580315

**Published:** 2025-06-16

**Authors:** YuMin Wang, Yi Ding, HaoLin Liu, ZhongYou Xia, Guoqiang Liao, ShiCheng Fan, JunXiong Li, JingBo Qin, PinYao Liang, Peng Gu, XiaoDong Liu, RuNan Dong

**Affiliations:** ^1^ The Department of Surgery, Shenzhen Longgang Second People’s Hospital, Shenzhen, China; ^2^ Department of Urology, The First Affiliated Hospital of Kunming Medical University, Kunming, Yunnan, China; ^3^ Department of Urology, The Third Affiliated Hospital of Wenzhou Medical University, Wenzhou, Zhejiang, China; ^4^ Department of Urology, Institute of Urology, West China Hospital, Sichuan University, Chengdu, Sichuan, China; ^5^ Department of Urology, Beijing Anzhen Nanchong Hospital, Capital Medical University and Nanchong Central Hospital, Nanchong, China

**Keywords:** cancer-associated fibroblasts (CAFs), tumor microenvironment (TME), single-cell RNA sequencing (scRNA-seq), risk score model, immunotherapy, drug sensitivity

## Abstract

**Background:**

Cancer-associated fibroblasts (CAFs) are essential components of the tumor microenvironment (TME), contributing to tumorigenesis, progression, and resistance to therapy. However, the functional diversity of CAF subpopulations and their role in tumor progression and patient prognosis remain poorly understood. This study aims to explore CAF heterogeneity and their functional roles in the TME using single-cell RNA sequencing (scRNA-seq) and multi-omics data analysis.

**Methods:**

scRNA-seq data were analyzed to cluster CAF subpopulations in the TME, with key genes identified through functional annotation. Differentially expressed genes were analyzed, and prognostic genes were selected via Cox and LASSO regression. A risk score model (RiskScore) was developed for survival prediction and immune therapy sensitivity evaluation. Core CAF genes were examined using siRNA interference, qPCR, and Western blotting. Drug sensitivity was assessed to explore the clinical relevance of these genes.

**Results:**

Four CAF subpopulations (CAF-0, CAF-1, CAF-2, CAF-3) were identified, revealing differences in key tumor-associated signaling pathways (e.g., MYC, WNT, TGF-β). Thirteen core genes related to prognosis were identified, and a RiskScore model was developed, showing significantly worse survival rates for high-risk patients (p < 0.001) and features of immune suppression, including increased M0 macrophage infiltration. Drug sensitivity analysis indicated that core genes (e.g., KLRB1, MAP1B) were linked to drug sensitivity, suggesting potential biomarkers for targeted therapy. Experimental validation showed that knockdown of the HIP1R gene significantly reduced tumor cell expression, confirming its critical role in tumor development.

**Conclusion:**

This study offers a comprehensive analysis of CAF heterogeneity and its impact on TME, patient prognosis, and drug sensitivity. The developed RiskScore model provides theoretical support for personalized treatment based on CAF-related genes, offering new insights into CAF-driven tumor progression and potential targets for precision oncology and immunotherapy.

## 1 Introduction

Bladder cancer (Bca) represents a prevalent malignancy impacting the genitourinary system globally. Epidemiological data indicate that approximately 430,000 new cases are diagnosed annually, with over 165,000 fatalities attributed to the disease each year (1). The pathogenesis and progression of bladder cancer are intricately linked to genetic and epigenetic modifications within cancer cells. Furthermore, the tumor microenvironment (TME), comprising immune cells, stromal components, and a variety of signaling molecules, significantly contributes to tumor proliferation, invasion, and therapeutic resistance. A comprehensive understanding of the complex interactions among these factors is essential for advancing diagnostic, prognostic, and therapeutic approaches.

TME comprises a complex and dynamic network of tumor cells, stromal cells, immune cells, and components of the extracellular matrix (ECM). Among the principal stromal cell types, cancer-associated fibroblasts (CAFs) are recognized as one of the most prominent and functionally versatile elements. These cells play a critical role in promoting tumor growth, invasion, and metastasis through various mechanisms. CAFs secrete a range of growth factors, cytokines, and chemokines that stimulate tumor cell proliferation and invasion (2, 3). Additionally, they actively engage in ECM remodeling by modifying its structure and biochemical composition, thereby facilitating tumor cell migration and contributing to the establishment of a supportive microenvironment for cancer progression. Elucidating the complex roles of CAFs and their interactions with other TME components may lead to the development of novel therapeutic strategies for bladder cancer (4, 5).

Nevertheless, CAFs demonstrate considerable heterogeneity, complicating a comprehensive understanding of their roles within the TME. This heterogeneity is evident in variations in their phenotype, function, and the signaling pathways they engage. As a result, delineating the precise contributions of CAFs to tumor biology remains a formidable challenge. Among the various subpopulations of CAFs, distinct functional differences are observed, with some subgroups potentially exhibiting opposing roles. While certain CAF subsets facilitate tumor invasion and metastatic progression (6), others may possess the capacity to suppress tumor advancement (7). Furthermore, the biological functions of these CAF subpopulations are intricately influenced by factors such as their cellular origin, the activation state of specific signaling pathways, and their interactions with tumor and immune cells (2, 7). The advent of advanced technologies, notably single-cell RNA sequencing (scRNA-seq), has furnished a robust methodology for the precise classification and characterization of CAFs within the TME. This technique has markedly enhanced our comprehension of the molecular attributes and functional diversity of CAFs, offering novel insights into their heterogeneity and prospective roles in cancer therapy.

Previous research has highlighted the substantial influence of CAFs on tumor progression (6, 8). However, the detailed phenotypic characteristics and functional distinctions among CAF subpopulations within various TMEs are not yet fully elucidated. Specifically, the complex interactions between CAFs, tumor cells, and immune components, along with their roles in processes such as tumor invasion, metastasis, and immune evasion, require more comprehensive study. Furthermore, the prognostic utility and functional significance of CAF marker genes in predicting patient outcomes, therapeutic responses, and the regulation of drug resistance remain incompletely understood. This study aims to explore the heterogeneous subpopulations of CAFs in both muscle-invasive and non-muscle-invasive BCa using single-cell analytical methodologies. The investigation will focus on elucidating the interactions between CAF subpopulations and the TME, thereby enhancing our understanding of the functional attributes and underlying mechanisms of pivotal CAF-associated genes. To achieve this, advanced bioinformatics techniques and experimental validations will be employed to dissect the molecular pathways and interactions that facilitate tumor progression and contribute to therapy resistanc. Moreover, this study aims to develop a multidimensional analytical framework by correlating the molecular characteristics of CAF subpopulations with clinical outcomes, immune therapy responses, and drug sensitivity in bladder cancer patients. The anticipated findings are expected to yield significant insights and novel methodologies for personalized treatment strategies and prognostic evaluations in both muscle-invasive and non-muscle-invasive BCa. Ultimately, this research has the potential to pave the way for innovative therapeutic strategies that specifically target distinct CAF subpopulations, thereby enhancing treatment efficacy and addressing drug resistance.

## 2 Materials and methods

### 2.1 Data collection and processing

This study leveraged scRNA-seq data from the GSE130001 dataset in the Gene Expression Omnibus (GEO) (9). Initial filtering criteria required that each gene be expressed in at least 3 cells, and each cell express a minimum of 250 genes. The Seurat R package’s PercentageFeatureSet function was used to assess mitochondrial gene and rRNA proportions. Further refinement involved setting thresholds where each cell expressed at least 6,000 genes, with a unique molecular identifier (UMI) count exceeding 100. After processing, 7,690 cells were retained for analysis.

For the prognostic analysis, RNA-seq datasets with comprehensive survival information were obtained from The Cancer Genome Atlas (TCGA) and GEO via the GDC portal, with a primary focus on the TCGA-BLCA cohort (n = 408, excluding normal tissues). Cases lacking survival or outcome data were excluded, resulting in 408 samples for subsequent investigation. Standardized gene expression profiles and corresponding clinical information from the GEO datasets GSE31684 and GSE13507 were utilized as validation cohorts (10, 11). Furthermore, data on single nucleotide variants (SNVs) and masked copy number variations (CNVs) were retrieved from TCGA for integrative analysis.

Based on literature, ten signaling pathways associated with cancer—cell cycle, HIPPO, MYC, NOTCH, NRF1, PI3K, TGF-β, RAS, TP53, and WNT—were chosen for further analysis, and gene expression profiles were evaluated across the datasets.

### 2.2 Definition of CAFs

Given the heterogeneity and complexity of cancer-associated fibroblasts (CAFs) within the tumor microenvironment, we identified CAFs based on well-established marker genes reported in the literature. Specifically, we selected ACTA2 (α-SMA), FAP, PDGFRB, and NOTCH3 as the primary markers (7, 12–14). CAFs were defined as cells expressing at least two of these markers above a predefined threshold in single-cell RNA sequencing data. Although POSTN has been reported in certain studies, it was excluded from our criteria due to insufficient expression in this dataset (15).

As a result, 715 CAFs were identified from a total of 7,690 cells, and were included in downstream analyses, including clustering, functional enrichment, and immune-related characterization.

To comprehensively characterize CAFs in bladder cancer, we reanalyzed scRNA-seq data using the Seurat package (16). Cells expressing more than 6,000 or fewer than 250 genes were removed, and log-normalization was applied to gene expression data. To correct batch effects across 21 samples, the FindIntegrationAnchors function was utilized.

Dimensionality reduction was conducted utilizing Uniform Manifold Approximation and Projection (UMAP), incorporating 15 principal components with a resolution parameter set to 0.2. Subsequent cell clustering was executed using the FindNeighbors and FindClusters functions, with dimensions set to 40 and a resolution of 0.2. Further dimensionality reduction was implemented through t-distributed stochastic neighbor embedding (t-SNE) via the RunTSNE function. Fibroblast populations were identified based on the expression patterns of four key marker genes: ACTA2, FAP, PDGFRB, and NOTCH3.

Fibroblasts underwent reclustering using the FindNeighbors and FindClusters functions, with t-SNE employed for reducing dimensions.Marker gene analysis across fibroblast clusters was performed using the FindAllMarkers function, applying thresholds of logFC = 0.5, min.pct = 0.35, and an adjusted p-value < 0.05 to identify cluster-specific marker genes. KEGG enrichment analysis was applied to the identified markers through the clusterProfiler package (17).

Additionally, the CNV characteristics of CAF clusters were analyzed using the CopyKAT R package (18), facilitating the differentiation between tumor and normal cells within each sample.

### 2.3 Identification of core CAF genes

The limma package was used to identify genes that were expressed differently between tumor and normal tissues, with selection criteria of a false discovery rate (FDR) < 0.05 and |log_2_(Fold Change)| > 1. Correlation analysis between DEGs and CAF clusters was subsequently conducted, and key CAF-associated genes were selected based on a p-value < 0.001 and a correlation coefficient (cor) > 0.4.

To identify genes associated with prognosis with a significance level of p < 0.05, a univariate Cox regression analysis was performed utilizing the survival package. Subsequently, LASSO Cox regression analysis was employed to further refine the selection of genes. This was followed by a multivariate Cox regression analysis using a stepwise approach. A risk score formula was subsequently derived as follows: RiskScore = ∑(Exp_i_ × b_i_), where Exp_i_ denotes the expression level of gene i, and b_i_ represents its corresponding coefficient in the multivariate Cox model. After standardizing the data to have a mean of zero, patients were categorized into high-risk and low-risk groups.

The predictive performance of the risk score model was evaluated using receiver operating characteristic (ROC) curve analysis, conducted with the timeROC package. Similar analyses were performed on the validation cohort to verify the robustness of the model.

### 2.4 Immune landscape analysis

The CIBERSORT algorithm (19)was used to assess the proportion of 22 immune cell subtypes in the TCGA cohort, which was employed to evaluate immune cell infiltration. Additionally, the ESTIMATE algorithm was used to calculate immune scores and stromal scores to further explore the TME.

### 2.5 Risk score and nomogram construction

To develop a nomogram for clinical application, both univariate and multivariate Cox regression analyses were conducted on clinicopathological features and risk scores. Variables with a p-value of less than 0.05 from the multivariate Cox model were included in the nomogram for predicting Bca prognosis, which was constructed utilizing the rms package (20). The model’s predictive accuracy was evaluated through calibration curve analysis, while its clinical utility and reliability were assessed using decision curve analysis (DCA).

### 2.6 Immune checkpoint inhibitor responsiveness

We downloaded transcriptomic data and matched clinical data from the IMvigor210 cohort, which included bladder cancer patients treated with the anti-PD-L1 drug (Atezolizumab) (21). Additionally, the GSE78220 cohort, which contains transcriptomic data from melanoma patients treated with anti-PD-1 checkpoint inhibitors (22), was also obtained to assess the potential value of the risk score in predicting responsiveness to immune checkpoint blockade (ICB).

### 2.7 Cell culture

Bladder cancer cell lines RT4 and 5637, as well as normal bladder epithelial cells SVHUC, were cultured in Dulbecco’s modified Eagle’s medium (DMEM), McCoy’s 5A, and Ham’s F-12K (HyClone, Logan, USA) with 10% FBS (Invitrogen, Carlsbad, CA, USA) at 37°C in a 5% CO2 incubator. U251R cells were induced by temozolomide at an initial concentration of 5 µM, with the final concentration increased to 640 µM, maintaining each concentration for one month. Logarithmically growing cells were selected for experiments.

### 2.8 qRT-PCR analysis

Total RNA was extracted from RT4 and 5637 cells utilizing the TRIzol reagent (Invitrogen), followed by reverse transcription into complementary DNA (cDNA) in accordance with the protocol provided by the mRNA reverse transcription kit (Roche). Quantitative real-time PCR (qRT-PCR) was conducted using the SYBR Green RNA Kit (Applied Biosystems, USA), strictly following the manufacturer’s instructions. Detailed PCR conditions and primer sequences are presented in **Table 1**. Relative mRNA expression levels were determined using the 2-ΔΔCq method, with GAPDH serving as the reference gene.

**Table 1:** Primer sequences and PCR conditions used in this study.

Gene	Sequence(5'→3')
HIPIR-F	CTGAAACCCAAGAGCCTAGATG
HIPIR-R	TGGTTCATCATGTCCTCAATCC

### 2.9 Western blotting analysis

After cell processing according to the outlined steps, cells from each group were collected, washed twice with PBS, and lysed using RIPA buffer supplemented with phosphatase inhibitors. Lysates were incubated on ice for 30 minutes, followed by ultrasonic disruption. Protein concentrations were quantified using the BCA method. Proteins that were denatured (60 µg per well) were separated using SDS-PAGE and then transferred to PVDF membranes. Membranes were treated with 5% skim milk for two hours and then exposed to primary antibodies overnight at 4°C. Following three washes with PBST, the membranes were exposed to secondary antibodies at room temperature for two hours.Chemiluminescence detection was conducted using an enhanced chemiluminescence kit following three additional PBST washes. Protein bands were quantified using ImageJ software. Details of primary antibodies are provided in **Supplementary Table S2**.

### 2.10 Human Protein Atlas database and IHC validation

The Human Protein Atlas (HPA) online database (https://www.proteinatlas.org/) (23) provided data for examining the expression levels of CAF-related marker genes in bladder cancer samples.

### 2.11 Cell transfection

The sequences used in this study are as follows: sh-HIP 5′-GCTGC TGGATGAACAGTTT-3′, NC-RNAi: 5′-GTGAAACCGGGTGCTTATT-3′. Cells were cultured in 6-well plates at a concentration of 3-5 × 10^4 cells/ml at 37°C for 16–24 hours until they reached 30-50% confluence. Cells were treated with lentivirus and infection-enhancing solution according to the manufacturer’s instructions. The medium was replaced after 16 hours to promote further culture.

### 2.12 Colony formation assay

RT4 and 5637 cells were placed in 6-well plates at a concentration of 1,000 cells per well and grown under different experimental conditions for a period of two weeks. Following incubation, cells were washed with PBS, fixed with methanol, and stained using 0.1% crystal violet. Colonies containing 50 or more cells were counted under a microscope.

### 2.13 Invasion assay

For invasion testing, 40 μL of BD Matrigel (Corning, USA) was applied to the insert membranes and incubated at 37°C for 1 hour to solidify. In the upper chamber of the insert, 50,000 cells were suspended in 500 μL of serum-free 1640, McCoy’s 5A. The inserts were placed in 24-well plates containing 750 μL of 1640, McCoy’s 5A supplemented with FBS. After 24 hours, cells that passed through the insert were fixed with 4% paraformaldehyde and stained with 0.05% crystal violet. Invasive cells were quantified under a microscope.

### 2.14 Flow cytometry detection of bladder cancer cell cycle

Cell cycle analysis was performed using PI staining. After treatment, cells were collected and washed twice with PBS. Cells were treated with **0.25% trypsin** to generate a single-cell suspension, fixed overnight with 70% ethanol, then washed and incubated with PBS solution containing RNase (10 µg/mL) at 37°C for 30 minutes. Next, PI staining (50 µg/mL) was applied, and cells were incubated at 4°C in the dark for 30 minutes. Cell cycle distribution was analyzed using a flow cytometer (BD FACSCanto II), and the proportions of cells in G0/G1, S, and G2/M phases were determined. Flow cytometry data were analyzed with FlowJo software and plotted for apoptosis rates and cell cycle distributions. Experiments were conducted at least three times, with the results reported as mean ± SD.Comparisons between groups were made using the t-test, with p < 0.05 considered statistically significant.

### 2.15 Statistical analysis

All statistical analyses utilized R software, version 3.6.3. Correlation matrices were derived through Pearson or Spearman correlation methods. The comparison of the two groups was conducted using the Wilcoxon test. Survival differences were analyzed with Kaplan-Meier curves and Log-rank tests. A p-value of < 0.05 was considered statistically significant.

## 3 Results

### 3.1 Screening CAFs in single-cell RNA sequencing samples

**Figure 1:**
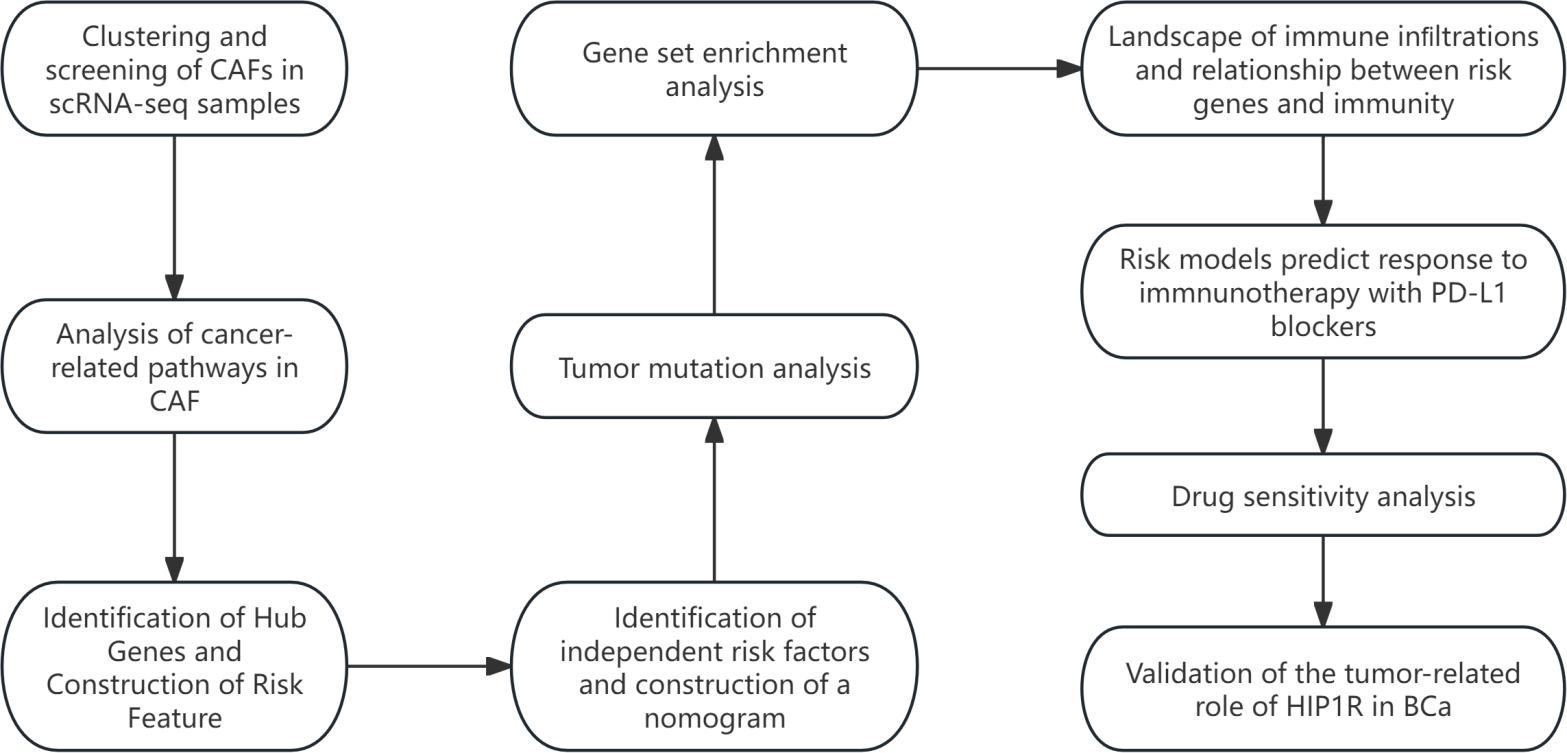
The workflow of this study.

**Figure 2:**
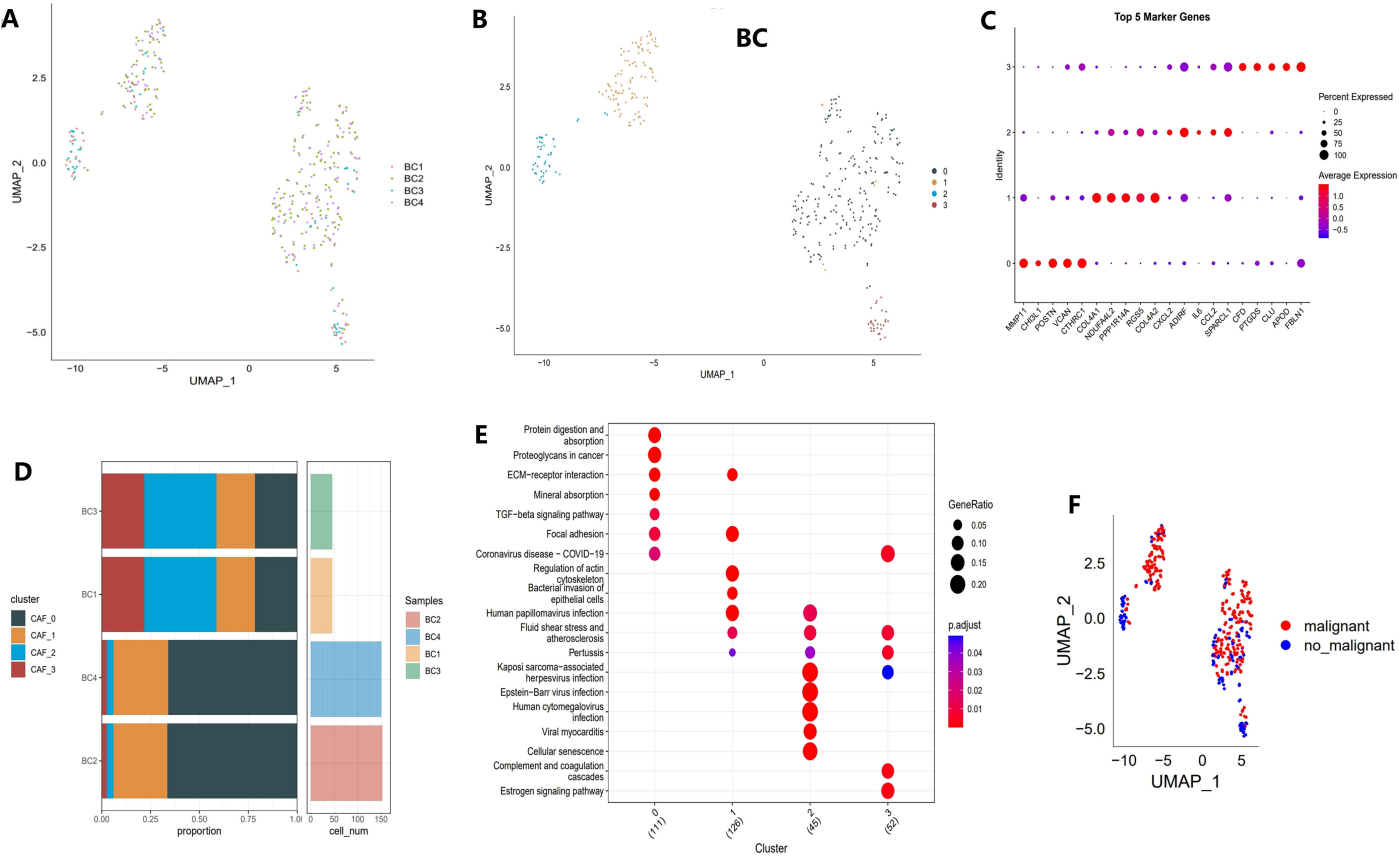
Identification of CAF clusters based on scRNA-seq data from BCa patients. **(A)** UMAP plot showing the distribution of 31 clusters and the expression of fibroblast marker genes. **(B)** UMAP plot displaying the distribution of five fibroblast clusters after reclustering. **(C)** Bubble plot illustrating the expression of the top four marker genes in each fibroblast subgroup. **(D)** Proportional distribution and cell count of fibroblast subgroups in tumor tissues. **(E)** KEGG pathway analysis of the four fibroblast subgroups. **(F)** UMAP plot depicting the distribution of malignant and non-malignant cells.

The study’s workflow is illustrated in **Figure 1**. Following an initial screening process, 7,690 cells were extracted from the single-cell RNA sequencing (scRNA-seq) data. Subsequent log normalization and dimensionality reduction procedures facilitated the identification of 20 subpopulations (**Supplementary Figures S1A, B**). Additional clustering and dimensionality reduction, utilizing four marker genes (ACTA2, FAP, PDGFRB, and NOTCH3), resulted in the delineation of four cancer-associated fibroblast (CAF) subpopulations (**Supplementary Figures S1C, D**). **Figure 2A** depicts the distribution of cells from each sample within the transcriptomic space. The findings revealed some overlap among samples; however, each sample exhibited distinct spatial distribution patterns, potentially indicative of gene expression variability across samples. Further clustering analyses identified functional states or cell-type differences within samples, culminating in the identification of four CAF populations for subsequent analysis (see **Figure 2B**).

Different clusters displayed specific marker gene expression profiles, which may be associated with particular functions or states (**Figure 2C**). **Figure 2D** shows that there were significant differences in the contribution of specific cell populations across samples. As shown in **Figure 2E**, distinct differences in functional pathway enrichment were observed among the cell populations. For example, pathways like “Protein Digestion and Absorption” and “TGF-beta Signaling Pathway” were significantly enriched in certain cell populations, potentially reflecting the specific functions or states of these groups. Moreover, some pathways were associated with known disease mechanisms, suggesting the biological importance of these cell groups. **Figure 2F** presents a UMAP projection comparing the distribution of malignant and non-malignant cells, revealing spatial separation with some degree of overlap. This overlap may indicate the transcriptomic heterogeneity of malignant cells while also suggesting shared gene expression patterns between malignant and non-malignant cells in specific contexts.

### 3.2 Expression of cancer-related pathways in CAFs

**Figure 3:**
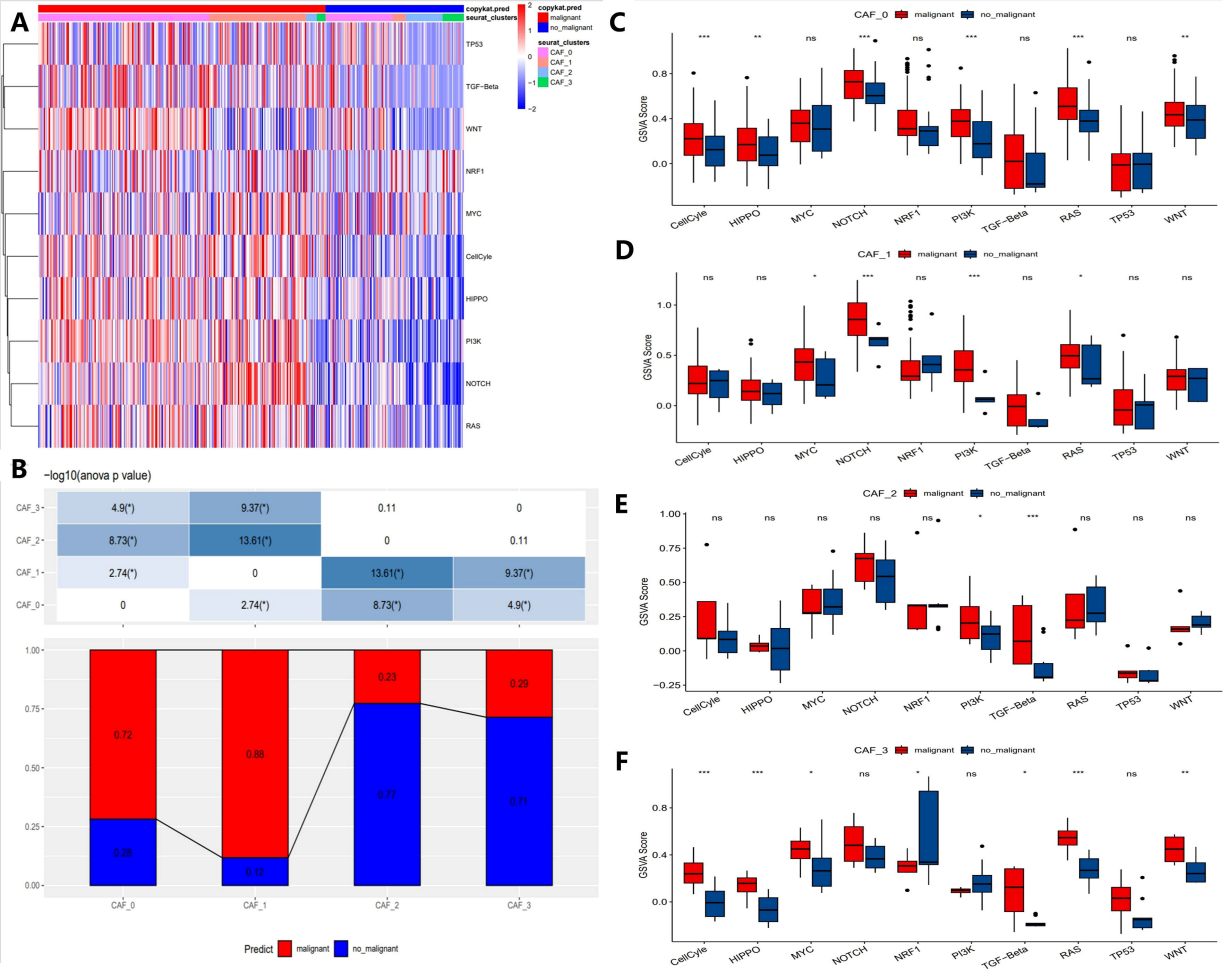
Characteristics of tumor-associated pathways in CAF clusters. **(A)** Heatmap showing the top 10 tumor-associated pathways enriched in CAF cells. **(B)** Proportional distribution of malignant cells across different CAF subgroups. **(C-F)** Comparison of GSVA scores for key genes in each CAF subgroup (CAF-0, CAF-1, CAF-2, and CAF-3). (“*” indicates p < 0.05, “**” indicates p < 0.01, “***” indicates p < 0.001, “ns” stands for “not significant”).

To elucidate the relationship between CAF populations and tumor progression, we analyzed the tumor-related pathway activities in different CAF subpopulations. The results showed that malignant cells exhibited higher expression of oncogenes (e.g., MYC, WNT, NOTCH1) in CAF subpopulations, while tumor suppressor genes (e.g., TP53) were significantly downregulated (**Figure 3A**). Notably, in the CAF-2 subpopulation, the differences between malignant and non-malignant cells were most pronounced, reflecting the complex heterogeneity of CAF subpopulations in tumor progression (**Figure 3B**). Furthermore, **Figures 3C–F** compare the Gene Set Variation Analysis (GSVA) scores of key genes across CAF subpopulations. Malignant cells consistently exhibited higher GSVA scores for MYC, WNT, and NOTCH1 compared to non-malignant cells, with the differences being most significant in CAF-0 and CAF-2 (p < 0.001). In contrast, some genes like TP53 showed no significant GSVA score differences across all subpopulations (ns). These results highlight the heterogeneity of CAF subpopulations in supporting malignant progression and emphasize the key roles of specific signaling pathways, such as MYC and WNT, in driving malignant progression.

### 3.3 Construction and validation of tumor risk prediction model based on differential genes and survival correlation analysis

**Figure 4:**
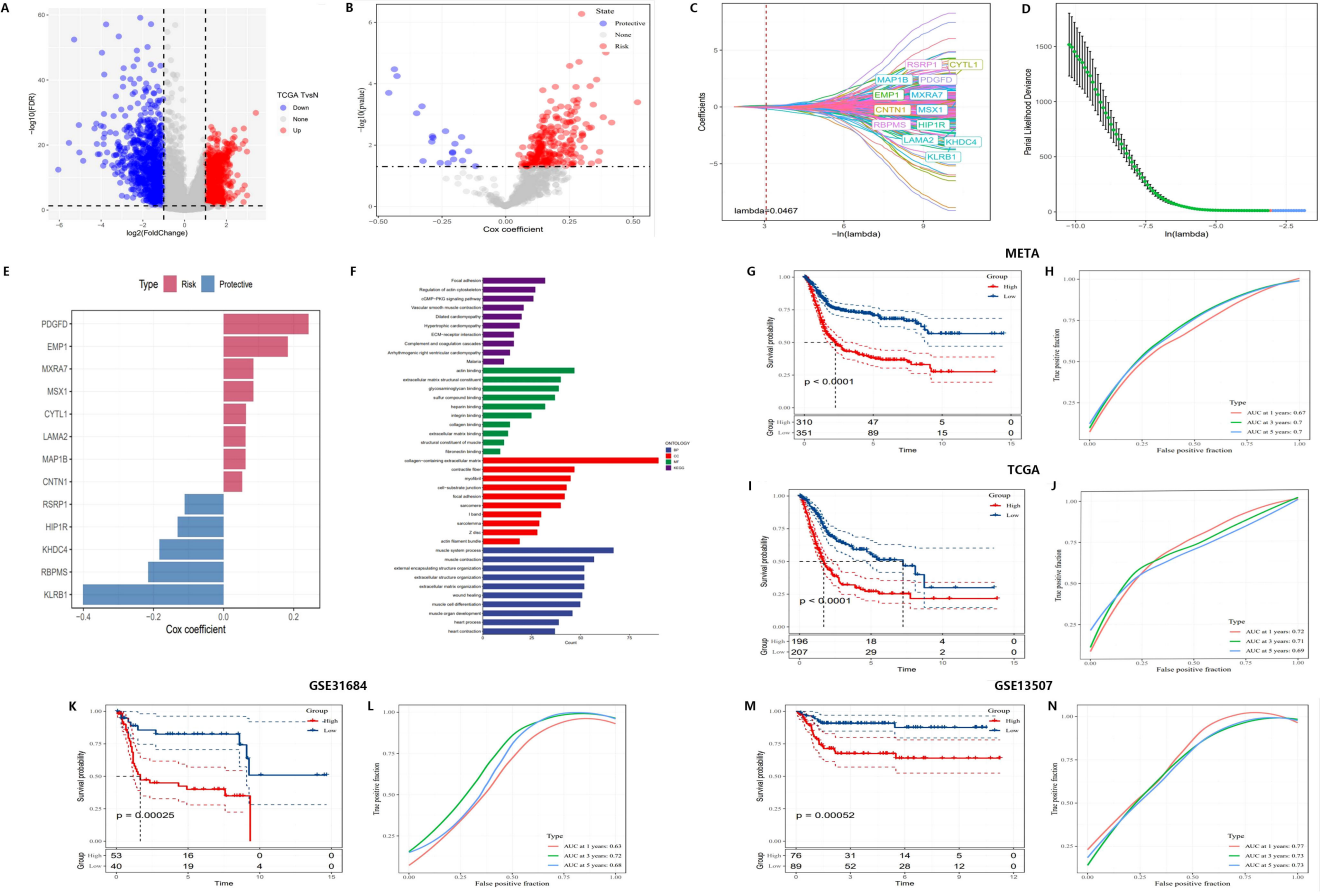
Construction of a novel risk signature based on CAF-related genes. **(A)** Volcano plot showing differentially expressed genes between tumor and normal samples in the TCGA cohort. **(B)** Volcano plot of prognosis-associated genes identified through univariate Cox regression analysis. **(C, D)** Lambda trajectory and distribution of each independent variable. **(E)** Cox coefficients of the selected genes. **(F)** Functional pathway enrichment analysis. **(G-N)** Kaplan-Meier survival curves and ROC curves of the risk signature in the META, TCGA, GSE31684, and GSE13507 cohorts.

To construct the risk score model, we initially identified differentially expressed genes (DEGs) between tumor and normal tissues, resulting in the identification of 1,622 DEGs, with 769 being upregulated and 853 downregulated (**Figure 4A**). Univariate Cox regression analysis was subsequently employed to identify genes significantly associated with patient survival, categorizing them as either risk or protective genes. Elevated expression of risk genes was associated with poor survival outcomes, indicating their potential involvement in critical pathways of tumor progression (**Figure 4B**). Further pathway enrichment analysis was conducted to elucidate the biological processes in which these differential genes may be involved. Pathways associated with risk genes were predominantly related to extracellular matrix remodeling, collagen metabolism, and cell adhesion, all of which are integral to tumor invasion and metastasis. Conversely, protective genes were linked to immune response and metabolic regulation pathways, suggesting their potential roles in maintaining cellular homeostasis and inhibiting tumor development (**Figure 4F**).

Additionally, LASSO regression was employed to identify 13 core genes, which were used to construct the final risk score model. Cross-validation results showed that at the optimal lambda value, the LASSO model effectively balanced predictive accuracy and model complexity (**Figures 4C, D**). The 13 genes were incorporated into the risk score formula as follows: RiskScore = 0.052 * CNTN1 expression + 0.063 * CYTL1 expression + 0.183 * EMP1 expression - 0.130 * HIP1R expression - 0.183 * KHDC4 expression + 0.062 * LAMA2 expression + 0.062 * MAP1B expression + 0.085 * MXRA7 expression - 0.110 * RSRP1 expression + 0.085 * MSX1 expression + 0.242 * PDGFD expression - 0.401 * KLRB1 expression - 0.215 * RBPMS expression. This model assigned risk scores to each sample, categorizing them into high-risk and low-risk groups. Cox regression further validated the survival prediction potential of these genes, with risk genes showing positive Cox coefficients, indicating that their elevated expression significantly increased the risk of mortality, while protective genes had negative coefficients, correlating with improved survival (**Figure 4E**).

For further systematic validation, Kaplan-Meier survival curves (**Figures 4G, I, K, M**) were generated using multiple datasets (META, TCGA, GSE31684, and GSE13507), confirming the applicability of the risk model. The results demonstrated that patients in the high-risk group had significantly lower survival rates compared to those in the low-risk group (p < 0.0001). The model exhibited consistent performance across different datasets, highlighting its stability and robustness in survival prediction.

**Figure 5:**
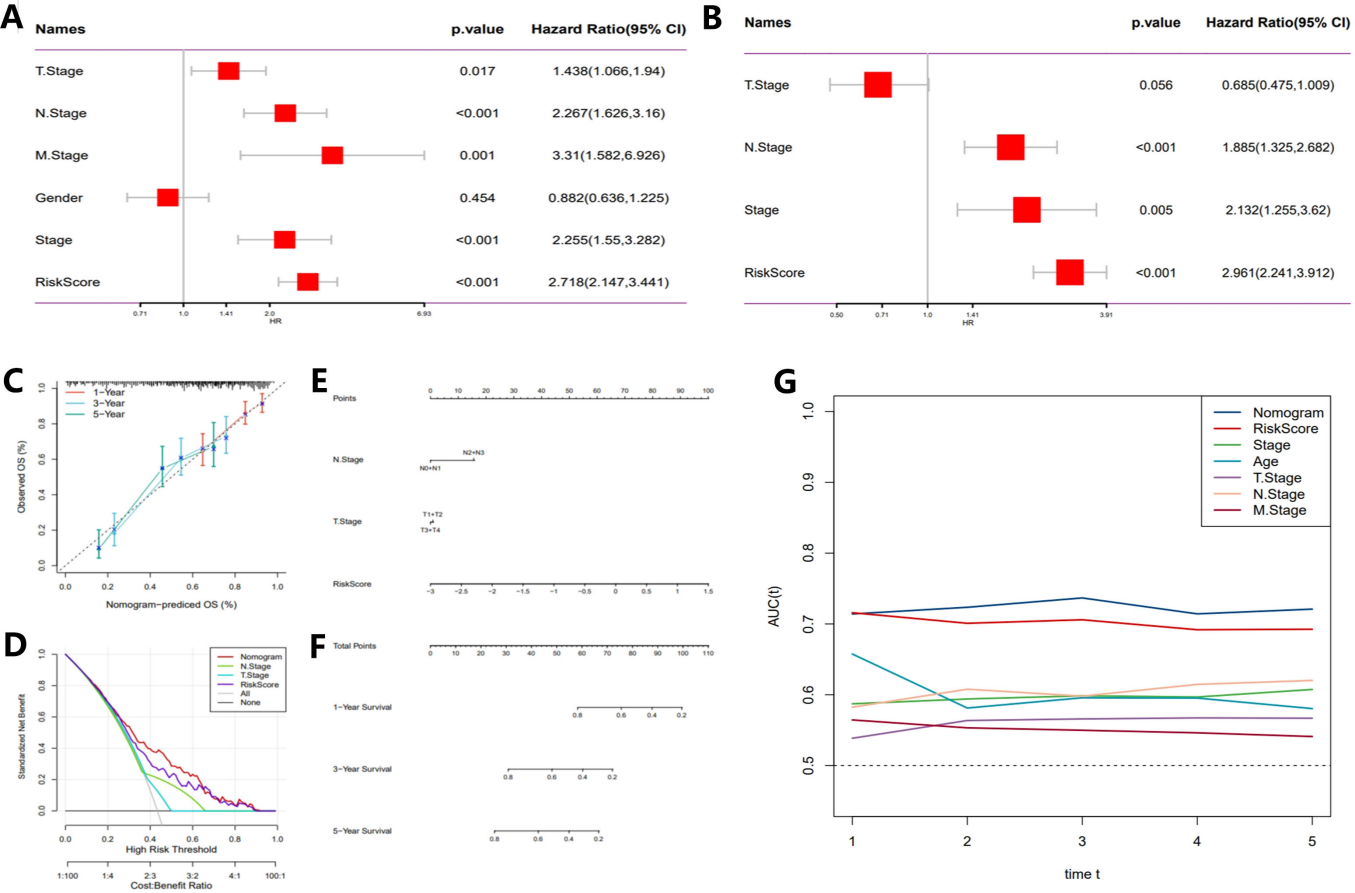
Development of a novel nomogram integrating the risk signature and multiple clinicopathological features. **(A)** Multivariate Cox regression analysis based on risk score and clinicopathological features. **(B)** Univariate Cox regression analysis based on risk score and clinicopathological features. **(C)** Calibration curves for predicting 1-, 3-, and 5-year survival. **(D)** Decision curve analysis (DCA) assessing the clinical net benefit of the risk score and nomogram model across different risk thresholds. **(E)** Nomogram incorporating the risk score and clinical staging. **(F)** Time-dependent ROC curves evaluating the predictive performance of the nomogram model. **(G)** Time-dependent concordance index (C-index) analysis showing the survival prediction ability of different variables at various time points.

ROC curves (**Figures 4H, J, L, N**) were employed to evaluate the predictive performance of the model, yielding AUC) values of 0.77, 0.78, and 0.75. These results further corroborate the model’s accuracy in forecasting survival outcomes at 3 and 5 years. Subsequent multivariate Cox regression analysis (**Figures 5A, B**) identified T-stage, N-stage, M-stage, and RiskScore as independent predictors of survival, with N-stage (Hazard Ratio [HR] = 2.267, p < 0.001) and M-stage (HR = 3.31, p = 0.001) exerting significant influence on patient mortality risk. The HR for the RiskScore was 2.718 (p < 0.001), underscoring its pivotal role in survival prediction. Nomogram models (**Figures 5C–E**) were developed by integrating T-stage, N-stage, and RiskScore to provide predictive tools for 1-year, 3-year, and 5-year survival, with predicted values demonstrating high concordance with actual observations, thereby validating the model’s clinical superiority (AUCs of 0.77, 0.78, and 0.75, **Figure 5F**). Finally, time-dependent C-index analysis revealed that the nomogram model consistently exhibited the highest C-index at each time point, indicating its moderate to good predictive capability predictive capability (**Figure 5G**).

These results provide reliable theoretical support for tumor prognosis assessment and personalized treatment strategies.

### 3.4 Mutation characteristics, signaling pathway impacts, and copy number variation analysis of tumor-related genes

We analyzed the SNV mutations of 13 key genes in the risk score model and found that 17.15% of samples showed mutations in these genes. Mutation types included missense mutations, nonsense mutations, frame-shift deletions, and splice site mutations (**Supplementary Figure S2A**). LAMA2, MAP1B, and HIP1R were among the genes with higher mutation frequencies (8%, 6%, and 5%, respectively), suggesting these mutations play a crucial part in tumor initiation and progression, and these genes could be potential driver genes or therapeutic targets. Further analysis of the co-occurrence and mutual exclusivity relationships between gene mutations revealed synergistic effects between MAP1B and HIP1R mutations, which might jointly participate in certain signaling pathways, while MSX1 and TP53 mutations exhibited mutually exclusive relationships, indicating their independent roles in different tumor mechanisms (**Supplementary Figure S2B**).

When analyzing the impact of mutations on core tumor signaling pathways, we found that classic pathways such as RTK-RAS, WNT, and PI3K were most significantly affected by mutations (**Supplementary Figure S2C**). These mutations might promote tumor cell proliferation and invasion by altering signaling networks. Further copy number variation (CNV) analysis showed that most genes had stable copy numbers, but genes like MAP1B and HIP1R exhibited significant CNV gains or losses in some samples (**Supplementary Figure S2D**). These CNVs may enhance or diminish gene function, impacting tumor progression. Overall, these results reveal the genetic heterogeneity of tumors and their potential pathogenic mechanisms, providing an important molecular foundation for targeted therapy and precision medicine.

### 3.5 Core genes and their relationship with immunity

**Figure 6:**
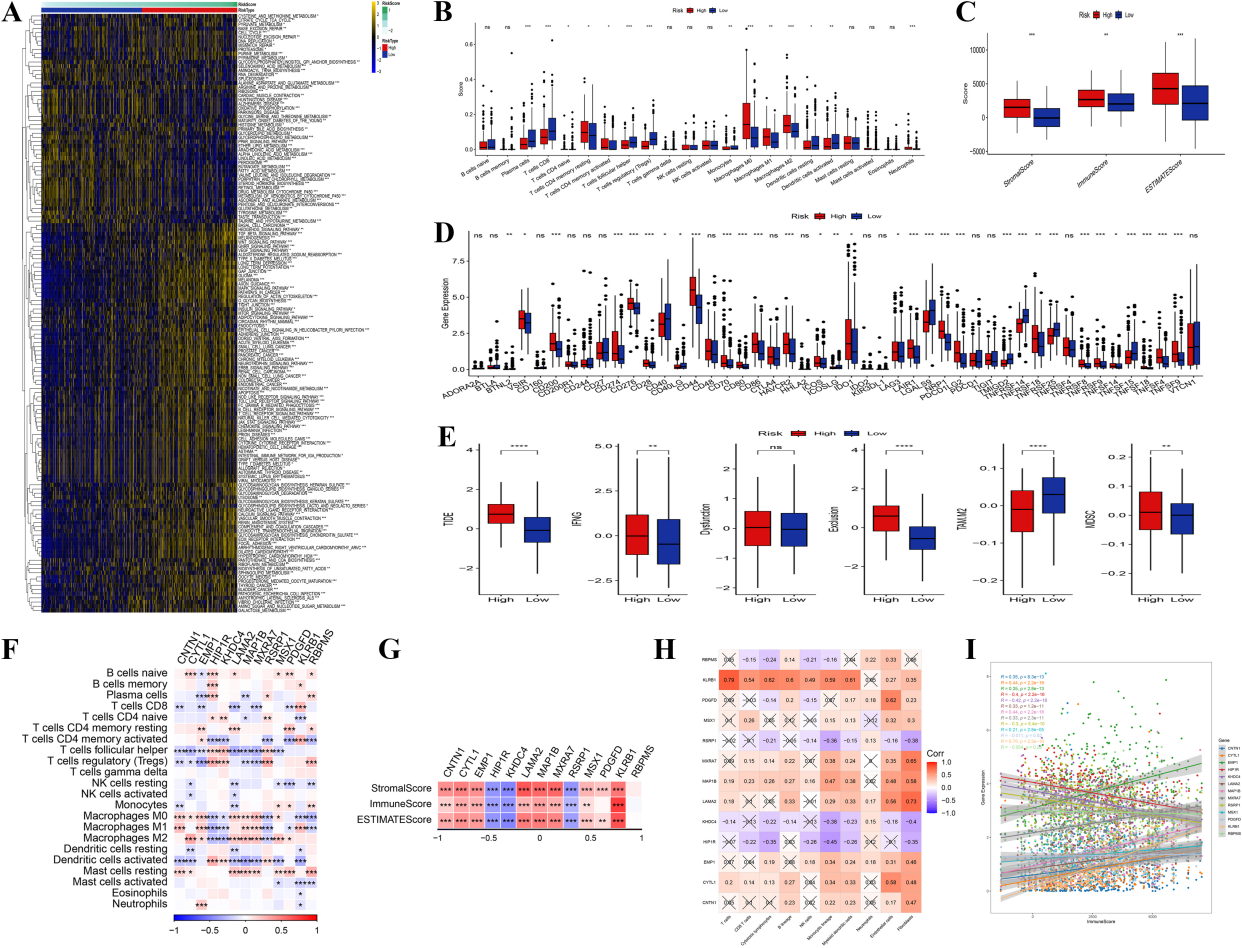
Immune infiltration analysis. **(A)** Heatmap of enriched pathways from DEGs between high- and low-risk groups. **(B)** Distribution differences of immune cell infiltration between high- and low-risk groups. **(C)** Comparison of immune score, stromal score, and ESTIMATE score between high- and low-risk groups. **(D, E)** Differences in signaling pathway and metabolic pathway activity between high- and low-risk groups. **(F)** Correlation analysis among different immune cells. The genes presented were identified through differential expression analysis between high- and low-risk groups, and are also constituents of the 13-gene risk score model. **(G)** Correlation analysis between core genes and immune score, stromal score, and ESTIMATE score. The genes presented were identified through differential expression analysis between high- and low-risk groups, and are also constituents of the 13-gene risk score model. **(H)** Correlation analysis between core genes and pro-tumor pathway genes in the high-risk group. **(I)** Correlation between core gene expression and pro-tumor characteristics in the high-risk group. (“*” indicates p < 0.05, “**” indicates p < 0.01, “***” indicates p < 0.001, “****” indicates p < 0.0001, “ns” stands for “not significant”).

In examining the relationship between tumor-related genes and signaling pathways, correlation heatmaps revealed significant positive associations between key genes (such as LAMA2, HPIPR, and CNTN1) and pathways associated with tumor-invasive phenotypes, including extracellular matrix remodeling and collagen formation (**Supplementary Figure S3A**). Gene expression heatmaps further indicated heterogeneity in the expression patterns of these critical genes across different samples (**Supplementary Figure S3B**). An analysis of TME characteristics in high-risk versus low-risk groups (**Figure 6A**), encompassing gene expression, immune cell infiltration, pathway activity, and metabolic features, demonstrated an enrichment of immune-suppressive cells (e.g., M0 macrophages) in the high-risk group, whereas anti-tumor immune cells (e.g., CD8+ T cells) were more prevalent in the low-risk group (**Figure 6B**). Additionally, the assessment of immune, stromal, and ESTIMATE scores revealed that stromal scores were significantly elevated in the high-risk group, while immune scores were higher in the low-risk group (**Figure 6C**).

Additionally, pathway and metabolic pathway activity analysis (**Figures 6D, E**) revealed increased activity of pro-tumor signaling pathways (e.g., TGF-beta) and elevated redox reactions in the high-risk group, indicating that these tumor cells support rapid proliferation through metabolic reprogramming. In contrast, the low-risk group displayed enhanced anti-tumor pathways (e.g., NK cell-mediated cytotoxicity) and glycolytic activity. The correlation analysis of immune cells indicated a positive relationship between immune-suppressive cells and pro-tumor signaling pathways, while anti-tumor immune cells were negatively correlated with suppressive signals (**Figure 6F**). Further investigation revealed that core genes (e.g., CNTN1, LAMA2) within the high-risk category, there was a positive correlation with stromal scores and a negative correlation with immune scores, suggesting that these genes promote tumor invasion and immune evasion by modulating the tumor stroma and inhibiting immune cell infiltration (**Figure 6G**). These core genes were significantly positively correlated with pro-tumor pathway genes, highlighting their synergistic role in high-risk tumor patients (**Figure 6H**). Overall, the high expression of core genes was strongly associated with enhanced immune suppression and stromal activity, suggesting their potential as biomarkers for poor prognosis in high-risk patients (**Figure 6I**). These findings highlight the role of the TME and core genes in tumor progression, suggesting molecular targets for therapeutic strategies in patients with high-risk tumors.

### 3.6 Risk score model and PD-L1 blockade immune therapy response

**Figure 7:**
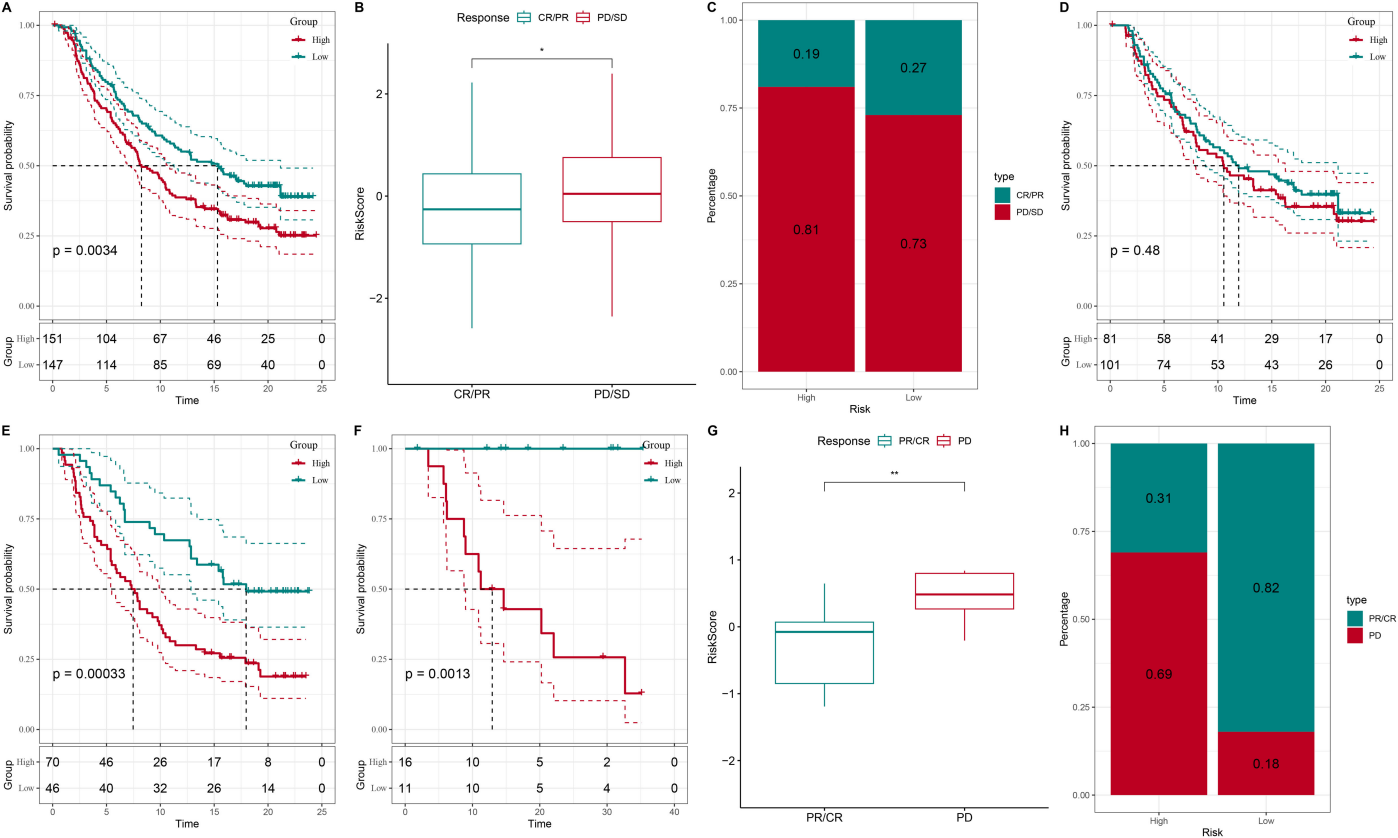
Correlation between risk score and response to PD-L1 immunotherapy. **(A)** Prognostic differences between risk subgroups in the IMvigor210 cohort. **(B, G)** Correlation analysis between risk score and treatment response. **(C, H)** Association between high- and low-risk groups and treatment response types. **(D)** Validation of the risk score model in an independent dataset. **(E, F)** Validation of the risk score in specific patient subsets and small sample populations. (“*” indicates p < 0.05, “**” indicates p < 0.01).

The association between risk scores and responses to PD-L1 immunotherapy was evaluated in the IMvigor210 and GSE78220 cohorts. Within the IMvigor210 cohort, comprising 348 patients, the administration of the anti-PD-L1 receptor blocker resulted in a spectrum of responses, including complete response (CR), partial response (PR), stable disease (SD), and progressive disease (PD). Kaplan-Meier survival analysis indicated that patients classified as high-risk experienced significantly reduced survival rates compared to their low-risk counterparts (p = 0.0034) (**Figure 7A**). Subsequent analyses (**Figures 7B, G**) revealed that patients achieving CR/PR had substantially lower risk scores than those with PD/SD.

Furthermore, our analysis revealed a correlation between risk stratification and treatment response categories. Within the high-risk cohort, 81% and 69% of responses were classified as PD or SD, whereas CR or PR constituted only 19% and 31% of the responses, respectively. Conversely, the low-risk cohort demonstrated a significantly greater prevalence of CR/PR responses, at 27% and 82%, respectively, while the incidence of PD/SD responses was reduced to 73% and 18% (**Figures 7C, H**). The risk score model underwent additional validation using an independent dataset. Despite the absence of statistically significant survival differences between the high-risk and low-risk groups in this dataset (p = 0.48) (**Figure 7D**), these findings indicate that the model’s applicability may be constrained by variations between datasets. Thus, the model needs to be optimized based on specific patient data and sample characteristics for clinical application. Finally, we validated the risk score in specific patient subsets and small sample groups. In these analyses, patients classified as high-risk had notably lower survival rates compared to those in the low-risk category (p = 0.00033 and p = 0.0013) (**Figures 7E, F**).

These results collectively demonstrate that the risk score can serve as a reliable prognostic biomarker and offer an effective reference for personalized therapy. The applicability of the model and its performance across different patient populations further provide robust theoretical support for precision medicine in clinical practice.

### 3.7 Correlation analysis of core gene expression with drug sensitivity

Exploring the connection between gene expression and drug sensitivity further revealed that genes might regulate drug responses (**Supplementary Figure S4**). Our results revealed correlation patterns between several genes (e.g., KLRB1, RBPMS, MAP1B) and specific drugs (e.g., Nelarabine, Copanlisib, Tamoxifen). High expression of some genes significantly enhanced sensitivity to certain drugs (e.g., KLRB1 was positively correlated with Nelarabine), while other genes reduced responses to specific drugs (e.g., MAP1B was negatively correlated with Tamoxifen). Additionally, RBPMS and MAP1B genes showed consistent negative correlations or complex positive-negative correlation patterns with multiple drugs’ sensitivities.

These results` provide valuable clues for exploring the potential mechanisms of gene-drug responses and suggest that these genes could serve as biomarkers or targets for drug sensitivity, providing guidance for personalized treatment strategies.

### 3.8 Validation of model gene HIP1R expression in target tissues

**Figure 8:**
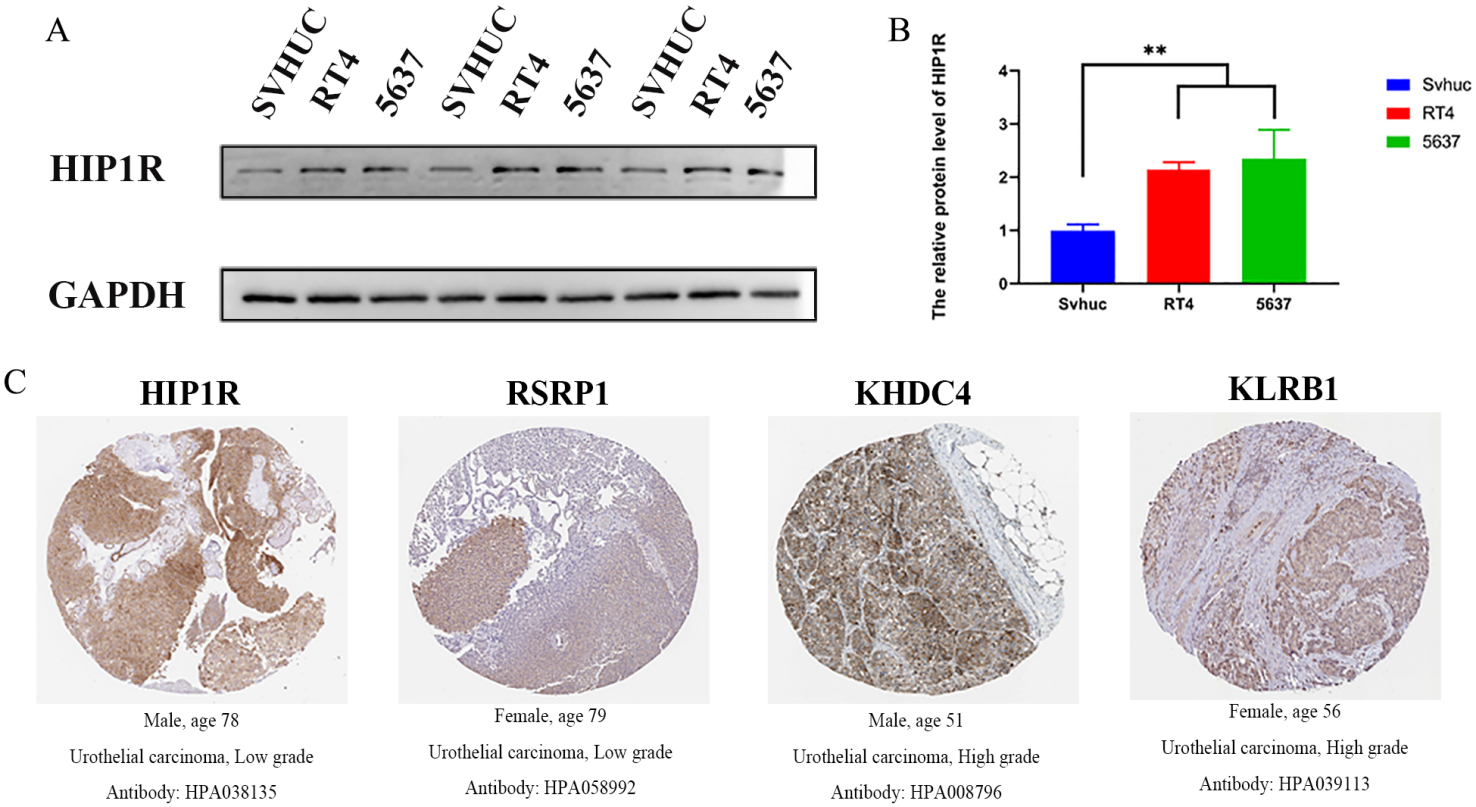
Expression of HIP1R in bladder cancer cell lines and urothelial carcinoma tissues. **(A)** Western blot analysis of HIP1R expression in normal bladder epithelial cells (SVHUC) and bladder cancer cell lines (RT4 and 5637). GAPDH was used as a loading control. **(B)** Quantification of HIP1R protein levels in SVHUC, RT4, and 5637 cells. Data are presented as mean ± SD, and statistical significance was determined using one-way ANOVA (P < 0.01). **(C)** Immunohistochemical (IHC) staining of HIP1R, RSRP1, KHDC4, and KLRB1 in urothelial carcinoma tissues of different grades. Representative images show positive staining in low-grade (HIP1R and RSRP1) and high-grade (KHDC4 and KLRB1) urothelial carcinoma tissues. (“**” indicates p < 0.01).

According to Western blot results, 5637 and RT4 cells exhibited higher HIP1R protein expression than SV-HUC cells, with RT4 cells expressing lower levels of HIP1R compared to 5637 cells (**Figures 8A, B**). Immunohistochemistry results also showed significant upregulation of HIP1R, RSRP1, KHDC4, and KLRB1 in BCa patient tissues (**Figure 8C**).

### 3.9 Impact of HIP1R knockdown on bladder cancer cell proliferation, migration, and clonal formation

**Figure 9:**
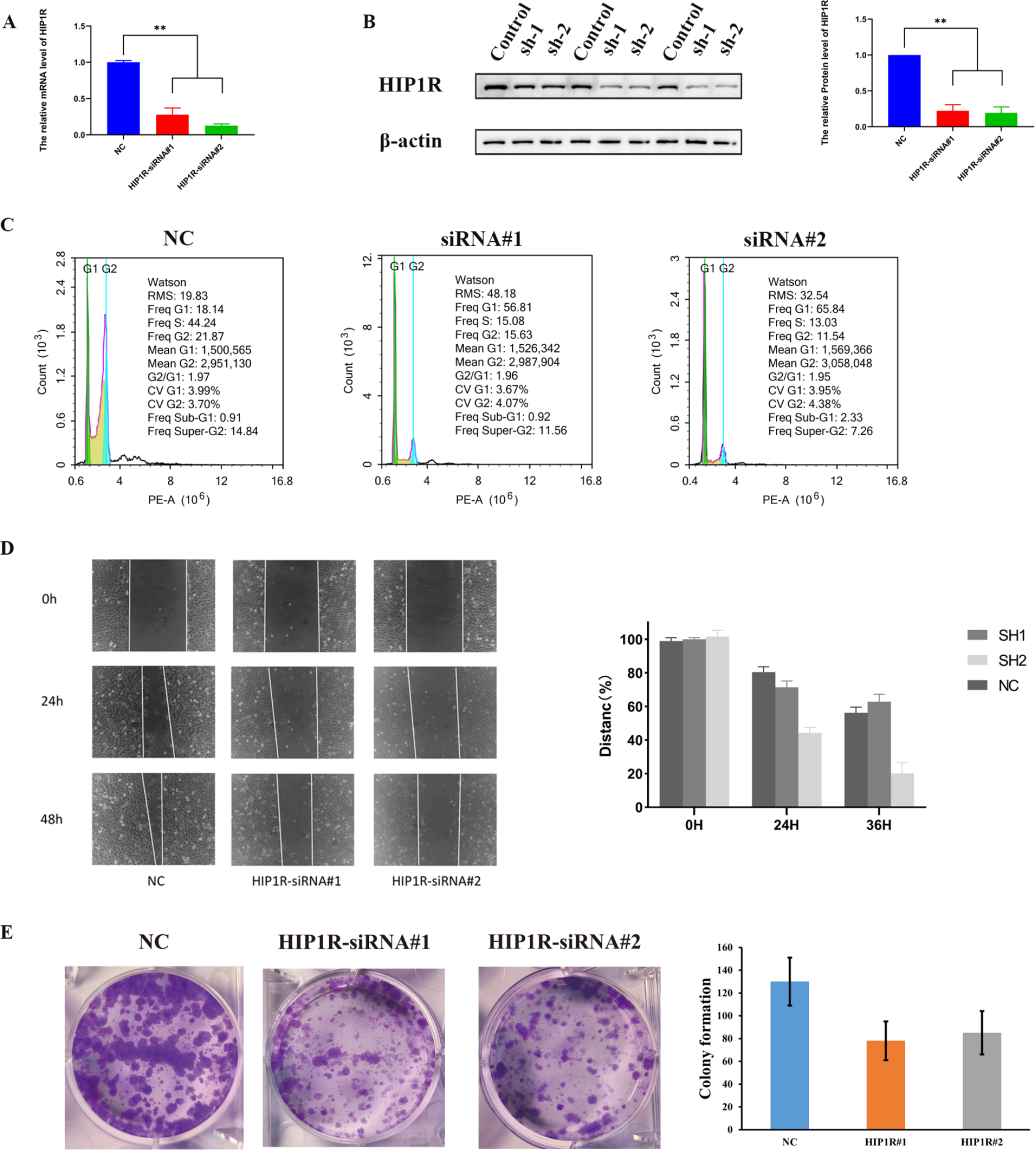
HIP1R knockdown inhibits bladder cancer cell proliferation, migration, and colony formation. **(A)** qRT-PCR analysis of HIP1R mRNA levels in control (NC) and HIP1R knockdown cells (HIP1R-siRNA#1 and HIP1R-siRNA#2). Data are presented as mean ± SD, P < 0.01. **(B)** Western blot analysis of HIP1R protein expression in control and knockdown groups. βroups. was used as a loading control. The quantification of HIP1R protein levels is shown on the right (P < 0.01). **(C)** Flow cytometry analysis of cell cycle distribution in NC and HIP1R knockdown groups. HIP1R knockdown increased the proportion of cells in the S phase while decreasing the G1 phase proportion. **(D)** Wound healing assay showing cell migration in NC and HIP1R knockdown groups at 0 h, 24 h, and 48h. The quantification of wound closure percentages is shown on the right. **(E)** Colony formation assay demonstrating reduced colony numbers in HIP1R knockdown groups compared to NC. The bar graph represents the quantification of colony numbers in different groups. (“**” indicates p < 0.01.).

We transfected HIP1R lentivirus into cells to elucidate its role in BCa. PCR and Western blot tests were used to validate the efficiency of the knockdown (**Figures 9A, B**). Compared to the control group, the siRNA-treated group had a higher percentage of cells in the G1 phase, according to flow cytometry analysis, while the S and G2 phases were significantly decreased, suggesting that HIP1R knockdown interfered with the normal cell cycle process (**Figure 9C**).

Subsequent experiments produced significant findings. In the wound healing assay, knockdown of HIP1R markedly diminished the migratory capacity of RT4 and 5637 cells, particularly at 24 and 48 hours, where the wound healing rate in the knockdown group was significantly lower than that of the control group (**Figure 9D**). Additionally, colony formation assays demonstrated a significant reduction in the clonal formation ability of RT4 and 5637 cells in the treatment group (**Figure 9E**), suggesting that HIP1R knockdown impedes BCa cell clonal formation. In summary, HIP1R is integral to the proliferation, migration, and clonal formation of BCa cells, and its knockdown significantly inhibits these processes.

## 4 Discussion

CAFs play a pivotal role in the TME, where they not only secrete cytokines, mediate extracellular matrix remodeling, and engage in direct cell-to-cell interactions to promote tumor cell proliferation, invasion, and metastasis, but also contribute significantly to immune evasion, drug resistance, and tumor recurrence (24). However, CAFs are not a homogeneous cell population, and their heterogeneity across different tumors and within the tumor itself complicates the full understanding of their regulatory mechanisms in tumor progression. Growing evidence indicates the importance of CAF-secreted factors and gene signatures linked to CAFs in predicting outcomes for bladder cancer patients (25). This study focuses on the functional heterogeneity of CAF subpopulations and their interactions with malignant cells in the TME. Through a systematic classification and functional analysis of CAFs using single-cell RNA sequencing data, we successfully identified four distinct CAF subpopulations—CAF-0, CAF-1, CAF-2, and CAF-3—and confirmed their unique spatial distribution and functional characteristics within the TME. Our results revealed significant differences in the distribution of malignant cells across the CAF subpopulations, with distinct heterogeneity in the activity levels of specific pro-cancer signaling pathways, such as MYC, WNT, TGF-β, and PI3K. For instance, the CAF-2 subpopulation exhibited the highest pro-cancer signaling activity, while the related signals in CAF-3 were weaker. The PI3K signaling pathway has been established as a therapeutic target in bladder cancer (26) and pathways such as MYC, WNT, TGF-β, and PI3K have been shown to play critical roles in bladder cancer progression (27–30). Furthermore, a significant positive correlation was observed between core genes, such as HPIPR and LAMA2, within the high-risk group and various pro-tumor pathway genes. This finding suggests that these core genes may play a pivotal role in high-risk tumor patients through synergistic interactions.

Meanwhile, to comprehensively elucidate the potential role and predictive value of characteristic genes in tumor progression and patient prognosis, we identified 13 core differentially expressed genes associated with tumor patient prognosis and developed a risk score model based on these genes. By integrating the risk score with clinical features (such as T-stage and N-stage), a nomogram model was created by us to estimate the 1-year, 3-year, and 5-year patient survival rates. Validation results showed that the predictive ability of the nomogram model outperformed individual risk scores or clinical indicators, demonstrating higher accuracy (AUC > 0.7). Furthermore, the model exhibited consistent results across different datasets (e.g., META and TCGA), indicating its moderate to good stability and generalizability.

In the evaluation of immune therapy, the risk score model demonstrated significant advantages. We observed that in high-risk tumor patients showed a poorer response to PD-L1 immune checkpoint inhibitors, while the CR rate was notably higher in the low-risk group. This suggests that the risk score model can function not only as a prognostic tool but also as a predictive marker for identifying patients who are more likely to benefit from immune therapy. Additionally, patients in the high-risk group exhibited significantly lower survival rates compared to those in the low-risk group, and the risk score was strongly correlated with the immune infiltration characteristics of the TME. Previous studies have shown that the interaction between CAFs and the tumor immune micro-environment (TIME) plays a crucial role in tumor progression (31). In our study, core genes in the high-risk group exhibited significantly higher expression levels, positively correlating with stromal scores and negatively correlating with immune scores. This suggests that these genes may promote tumor invasion and immune evasion by modulating tumor stromal components and suppressing immune cell infiltration. Further GSVA analysis revealed the potential roles of different CAF subpopulations in regulating tumor cell growth, modulating immune responses, and driving metabolic reprogramming. CAFs have been shown to enhance tumor cell growth and reduce sensitivity to immune therapy in various malignant tumors (32–35).

Immune cells present within tumors play a pivotal role in modulating the anti-tumor immune response within the TME. CAFs engage with these immune cells to establish an immune-suppressive TME, thereby facilitating tumor cells’ evasion of immune surveillance (36). Notably, our study demonstrated a marked increase in pro-tumor immune cells, such as M0 macrophages, in the high-risk group, whereas anti-tumor immune cells, including activated CD8+ T cells, were significantly more prevalent in the low-risk group.

Furthermore, previous studies have demonstrated that tumor-associated macrophage (TAM) polarization/activation and CAF induction/recruitment are influenced by tumor-derived molecules, such as IL-6 (37, 38). The study’s findings shed light on the significance of the TME and core genes in tumor development, pointing to potential molecular targets and clinical strategies for designing targeted therapies for high-risk tumor patients.

Additionally, the study further integrated drug sensitivity data to analyze the correlation between core genes and specific drug sensitivities. The results revealed significant associations between KLRB1 and MAP1B expression and the sensitivity to several drugs. High expression of KLRB1 significantly enhanced sensitivity to Nelarabine and Cytarabine. Similarly, MAP1B exhibited a positive correlation with Simvastatin and a negative correlation with Tamoxifen, suggesting that MAP1B may play a regulatory role through distinct drug-specific mechanisms. Furthermore, the RBPS gene showed a negative correlation pattern with multiple drugs, such as Carmustine and Copanlisib. These results indicate that the expression levels of certain genes may directly affect tumor cell responses to specific drugs, providing a foundation for exploring these genes as biomarkers for drug sensitivity or therapeutic targets.

However, despite the important findings, several limitations remain. First, the heterogeneity of data sources could affect the generalizability of the model. For example, differences in patient characteristics and clinical treatment regimens across various databases may lead to inconsistencies in model predictions. Second, the experimental validation covered only a subset of the core genes (e.g., HIP1R, KLRB1, and MAP1B), and the function of other key genes still requires further investigation. Lastly, while the drug sensitivity analysis identified some gene-drug correlations, the underlying mechanisms remain unclear. Future research should focus on expanding the sample size, integrating data from multiple centers, and enhancing the clinical relevance of the model. Additionally, further investigation into the molecular mechanisms of core genes within the TME, particularly their roles in immune regulation and metabolic reprogramming, will provide deeper insights to support the development of novel targeted therapies and personalized treatment strategies. By integrating multi-omics data and functional validation, further advancement of CAF-related gene research will contribute to the clinical translation of these findings.

## 5 Conclusion

This study identifies the heterogeneity of CAFs and their roles in the TME, revealing four CAF subpopulations and 13 core genes for predicting patient prognosis and treatment sensitivity. The risk score model and experimental validation show that CAF core genes are closely linked to survival, immune micro-environment, and may serve as biomarkers for drug sensitivity and potential therapeutic targets. These findings provide crucial insights for understanding CAF mechanisms in tumors and their application in personalized treatment strategies.

## Data Availability

The datasets presented in this study can be found in online repositories. The names of the repository/repositories and accession number(s) can be found in the article/**Supplementary Material**.

## Supplementary material

Supplementary Figure 1 UMAP clustering and marker gene expression in single-cell RNA sequencing analysis. **(A)** UMAP visualization of single-cell transcriptomic data, with cells grouped into 20 clusters and colored accordingly. Each cluster represents a distinct cell population within the tumor micro-environment. **(B)** Expression patterns of marker genes ACTA2, FAP, PDGFRB, and NOTCH3 across different cell clusters. The color intensity represents the expression level, highlighting specific cell populations. **(C)** Refined clustering of cells into four major groups (0-3) based on UMAP representation. **(D)** Marker gene expression in the refined cell clusters, showing distinct expression patterns of ACTA2, FAP, PDGFRB, and NOTCH3, indicating functional specialization within the tumor micro-environment.

Supplementary Figure 2 SNV mutation landscape of the 13 genes in the risk score model. **(A)** Mutation profile based on the TCGA cohort. **(B)** Analysis of co-occurrence and mutual exclusivity relationships among key gene mutations. **(C)** Impact of mutations on core tumor signaling pathways. **(D)** CNV characteristics of key genes.

Supplementary Figure 3 Gene-pathway correlation and hierarchical clustering analysis of gene expression in tumor samples. **(A)** Heatmap showing the correlation between key genes (HIP1R, KHDC4, RBPMS, MAP1B, etc.) and various biological pathways. The color gradient represents correlation values, with red indicating positive correlation and blue indicating negative correlation. Asterisks denote statistical significance (*P < 0.05, **P < 0.01, **P < 0.001). **(B)** Hierarchical clustering heatmap illustrating gene expression patterns across different samples. Each row represents a KEGG pathway, and each column represents a sample. The color scale indicates the expression level, with red representing high expression and blue representing low expression. Genes such as HIP1R, KHDC4, RSRP1, and LAMA2 show differential expression across samples, suggesting potential involvement in tumor progression.

Supplementary Figure 4 Correlation analysis between gene expression and drug sensitivity.
