# Supplementary figures and images for: Therapeutic implications of cancer-associated fibroblast heterogeneity: insights from single-cell and multi-omics analysis
Source: Front Immunol. 2025 Jun 16;16:1580315. doi: 10.3389/fimmu.2025.1580315 (PMC12206818; doi:10.3389/fimmu.2025.1580315)

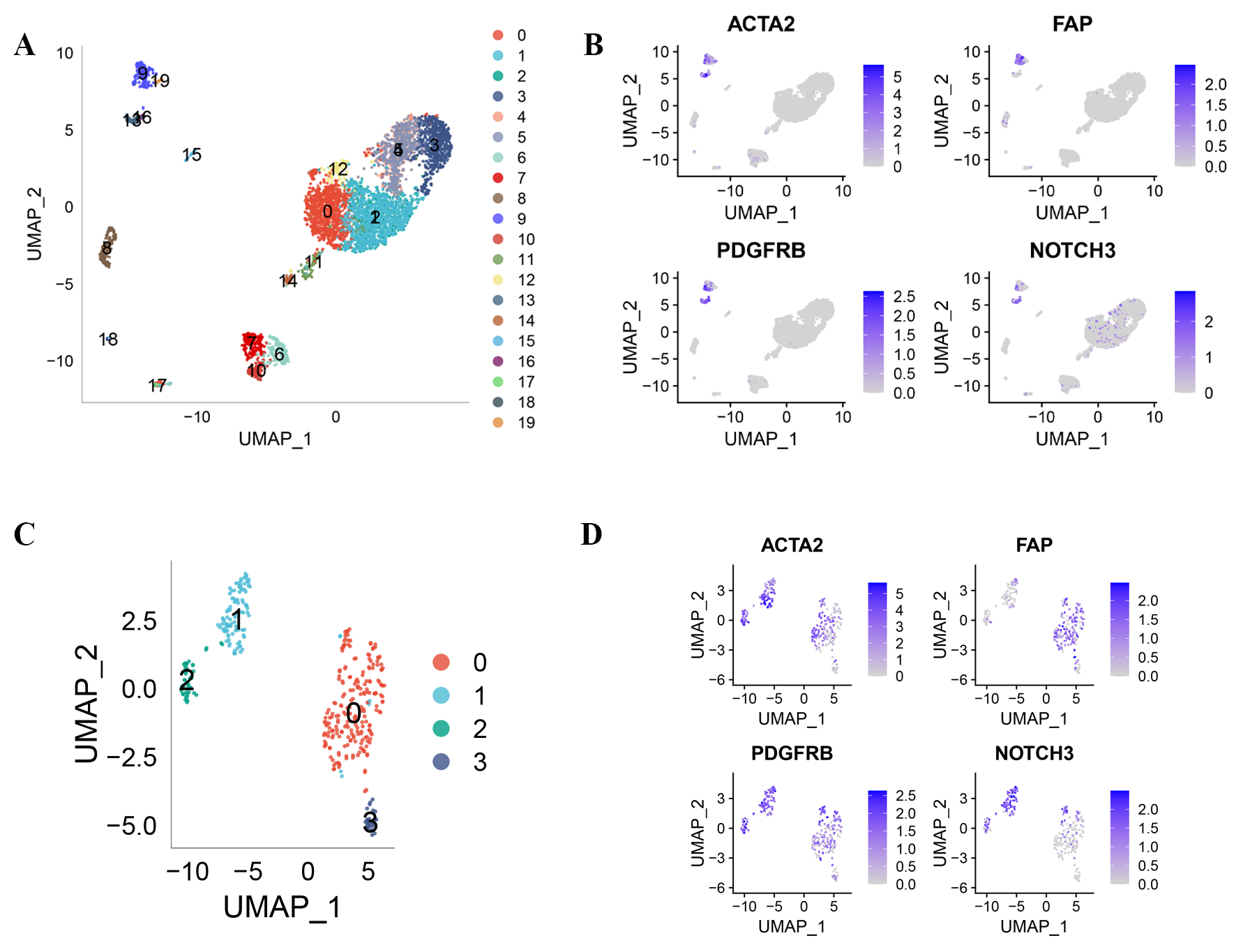

Supplement: Supplementary Figure 1 — UMAP clustering and marker gene expression in single-cell RNA sequencing analysis. (A) UMAP visualization of single-cell transcriptomic data, with cells grouped into 20 clusters and colored accordingly. Each cluster represents a distinct cell population within the tumor micro-environment. (B) Expression patterns of marker genes ACTA2, FAP, PDGFRB, and NOTCH3 across different cell clusters. The color intensity represents the expression level, highlighting specific cell populations. (C) Refined clustering of cells into four major groups (0-3) based on UMAP representation. (D) Marker gene expression in the refined cell clusters, showing distinct expression patterns of ACTA2, FAP, PDGFRB, and NOTCH3, indicating functional specialization within the tumor micro-environment. [file Image1.tif]

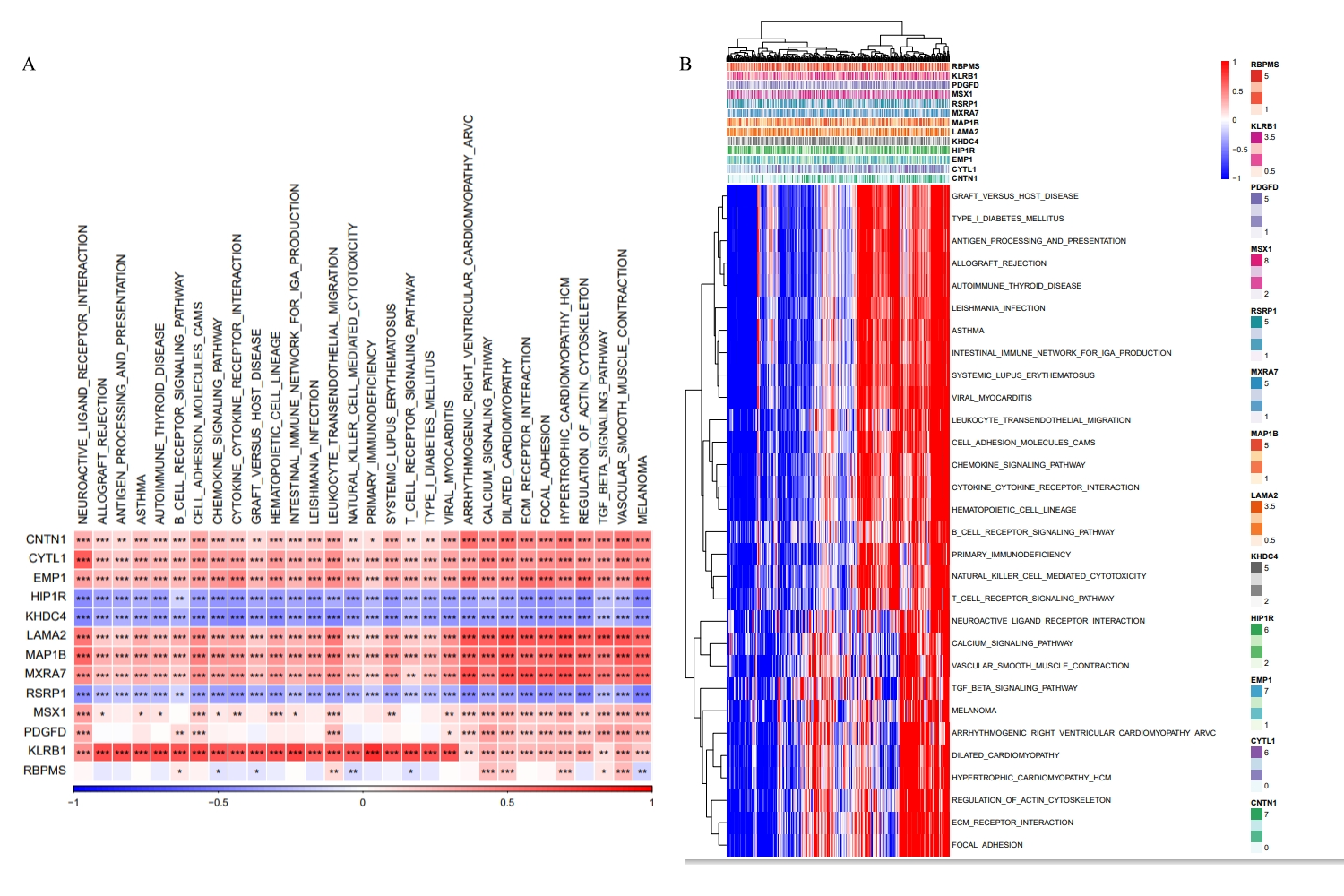

Supplement: Supplementary Figure 2 — SNV mutation landscape of the 13 genes in the risk score model. (A) Mutation profile based on the TCGA cohort. (B) Analysis of co-occurrence and mutual exclusivity relationships among key gene mutations. (C) Impact of mutations on core tumor signaling pathways. (D) CNV characteristics of key genes. [file Image2.tif]

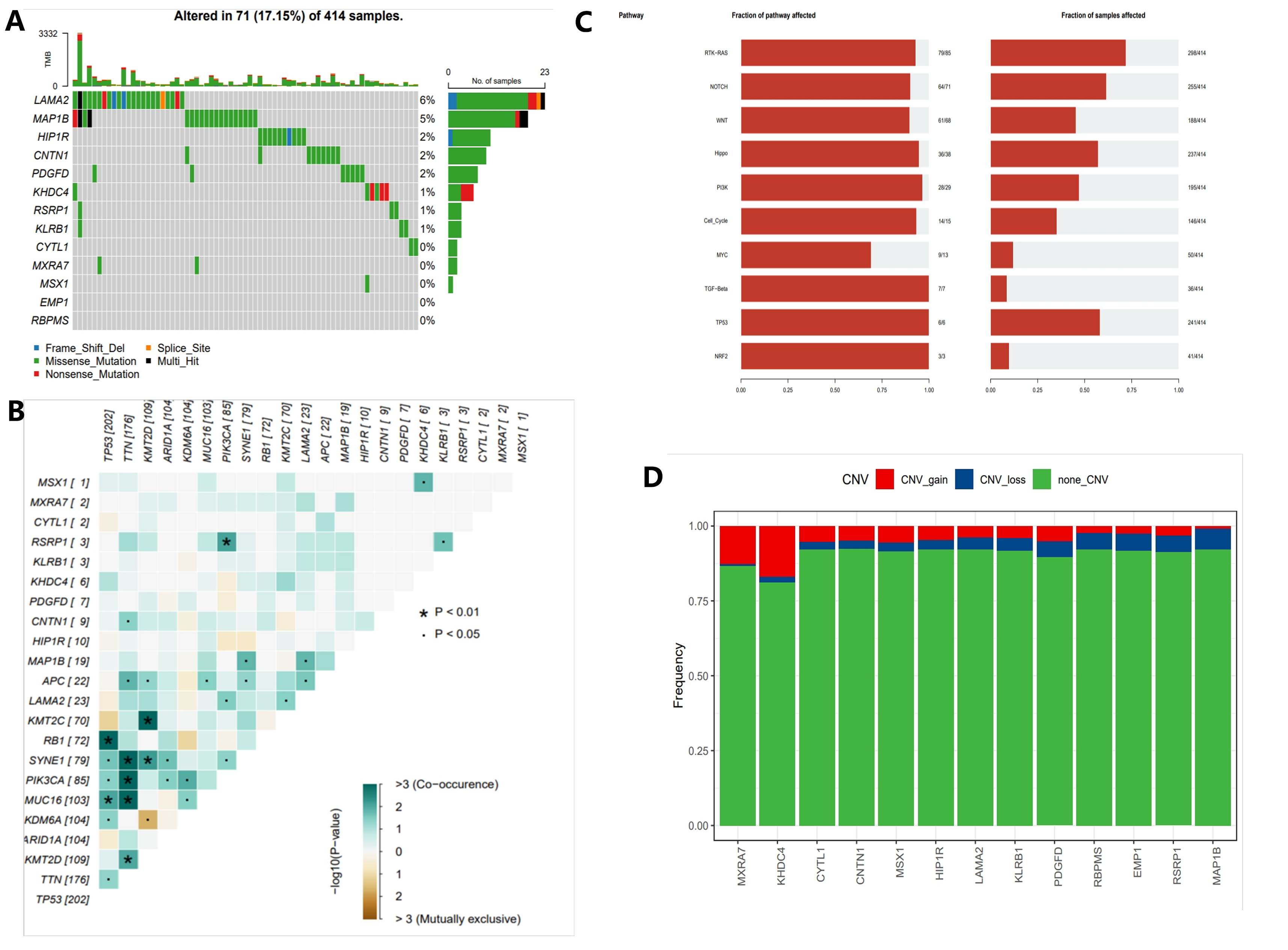

Supplement: Supplementary Figure 3 — Gene-pathway correlation and hierarchical clustering analysis of gene expression in tumor samples. (A) Heatmap showing the correlation between key genes (HIP1R, KHDC4, RBPMS, MAP1B, etc.) and various biological pathways. The color gradient represents correlation values, with red indicating positive correlation and blue indicating negative correlation. Asterisks denote statistical significance (*P < 0.05, **P < 0.01, **P < 0.001). (B) Hierarchical clustering heatmap illustrating gene expression patterns across different samples. Each row represents a KEGG pathway, and each column represents a sample. The color scale indicates the expression level, with red representing high expression and blue representing low expression. Genes such as HIP1R, KHDC4, RSRP1, and LAMA2 show differential expression across samples, suggesting potential involvement in tumor progression. [file Image3.tif]

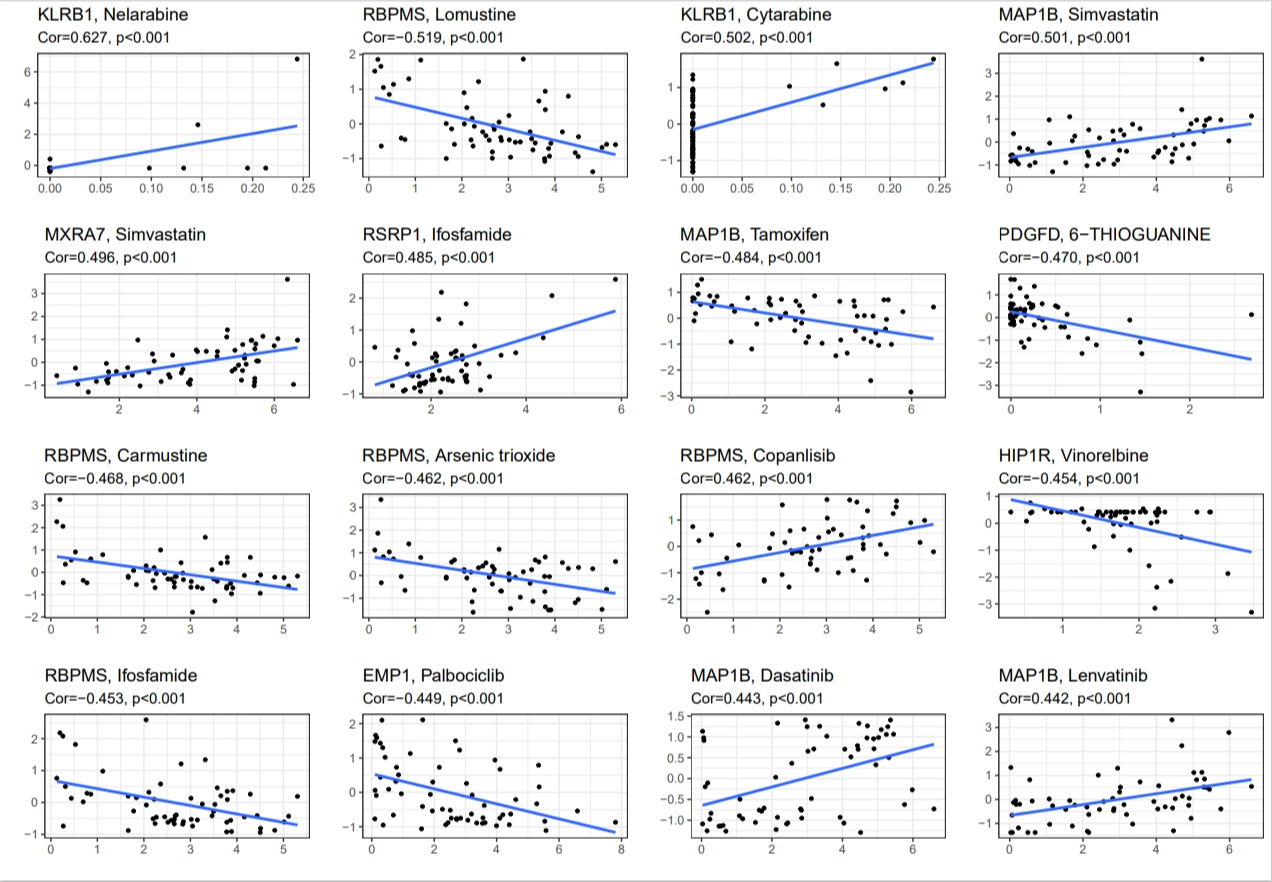

Supplement: Supplementary Figure 4 — Correlation analysis between gene expression and drug sensitivity. [file Image4.tif]
