# Supplementary material for: Therapeutic implications of cancer-associated fibroblast heterogeneity: insights from single-cell and multi-omics analysis
Source: Front Immunol. 2025 Jun 16;16:1580315. doi: 10.3389/fimmu.2025.1580315 (PMC12206818; doi:10.3389/fimmu.2025.1580315)
Supplement: Supplementary file 5 [file Table1.doc]

| Primary antibody | Company | Molecular weight | Article no | Dilutions(WB) |
| --- | --- | --- | --- | --- |
| HIP1R Monoclonal antibody | Proteintech | 120 kDa | 68403-1-IG | 1:1000 |
| GAPDH Monoclonal antibody | Proteintech | 36 kDa | 60004-1-IG | 1:1000 |
